# Clinical virtual simulation: predictors of user acceptance in nursing education

**DOI:** 10.1186/s12909-024-05154-2

**Published:** 2024-03-16

**Authors:** José Miguel Padilha, Patrício Costa, Paulino Sousa, Ana Ferreira

**Affiliations:** 1 Present Address: Nursing School of Porto, CINTESIS@RISE, Porto, Portugal; 2 Faculty of Medicine, University of Porto, Porto, Portugal; 3 Life and Health Sciences Research Institute (ICVS), School of Medicine, University of Minho, Braga, Portugal; 4 ICVS/3B’s - PT Government Associate Laboratory, Braga/Guimarães, Portugal; 5 Faculty of Psychology and Education Sciences, University of Porto, Porto, Portugal; 6 Nursing School of Porto, CINTESIS@RISE, Porto, Portugal; 7 CINTESIS@RISE, FMUP-MEDCIDS, Porto, Portugal

**Keywords:** Virtual patients, Clinical virtual simulation, Education, Simulation, Technology acceptance model, Nursing education

## Abstract

**Background:**

Using virtual patients integrated in simulators expands students’ training opportunities in healthcare. However, little is known about the usability perceived by students and the factors/determinants that predict the acceptance and use of clinical virtual simulation in nursing education.

**Objectives:**

To identify the factors/determinants that predict the acceptance and use of clinical virtual simulation in learning in nursing education.

**Methods:**

Observational, cross-sectional, analytical study of the use of clinical virtual simulation in nursing to answer the research question: What factors/determinants predict the acceptance and use of a clinical virtual simulator in nursing education? We used a non-probabilistic sampling, more specifically a convenience sample of nursing degree students. The data were collected through a questionnaire adapted from the Technology Acceptance Model 3. In technology and education, the Technology Acceptance Model is a theoretical model that predicts the acceptance of the use of technology by users.

**Results:**

The sample comprised 619 nursing students, who revealed mean values of perceived usefulness (M = 5.34; SD = 1.19), ease of use (M = 4.74; SD = 1.07), and intention to use the CVS (M = 5.21; SD = 1.18), in a Likert scale of seven points (1—the worst and 7 the best possible opinion).

This study validated the use of Technology Acceptance Model 3 adapted and tested the related hypotheses, showing that the model explains 62% of perceived utility, 32% of ease of use, and 54% of intention to use the clinical virtual simulation in nursing by nursing students. The adequacy of the model was tested by analysis of the direct effects of the relationships between the internal constructs (PU-BI, *β* = 0.11, *p* = 0.012; PEOU-BI, *β* = -0.11, *p* = 0.002) and the direct relations between some of the constructs internal to the Technology Acceptance Model 3 and the external determinants Relevance for learning and Enjoyability.

In the proposed model, the external constructs that best predicted perceived usefulness, ease of use, and behaviour intention to use the clinical virtual simulation in nursing were Relevance for learning and Enjoyability.

**Conclusions:**

These study results allowed us to identify relevance for learning and enjoyability as the main factors/determinants that predict the acceptance and use of clinical virtual simulation in learning in nursing.

**Supplementary Information:**

The online version contains supplementary material available at 10.1186/s12909-024-05154-2.

## Introduction

The rapid transformation of society in recent decades and the technical and scientific advancements in health sciences continually challenge higher education institutions (HEIs) to innovate, develop, and implement new pedagogical methodologies that guarantee up-to-date and quality training. Translating knowledge into clinical practice has become one of the main challenges of the first decades of the twenty-first century in research and higher education in health [1, 2]. The higher education institutions have been systematically confronted with the difficulty of teaching central and structuring concepts of clinical practice and difficulty in translating these concepts into clinical practice [1, 2]. Since the 1950s, multiple pedagogical strategies have been developed and implemented, such as Problem-Based Learning. These strategies intend to help students develop cognitive, instrumental, and attitudinal skills (Knowledge, Skills, Attitudes), among others, as structuring elements to ensure the quality and safety of their clinical practice. Quality and safety in clinical practice are associated with intrinsic and extrinsic factors for healthcare professionals. Of the intrinsic factors, clinical decision-making skills stand out. The development of clinical decision-making skills in healthcare students is one of the biggest challenges posed to higher education institutions teachers [3]. This becomes more evident in pre-graduate training with students without clinical experience. In addition, from a student's point of view, this is also one of the main challenges faced. Learning in the healthcare field implicates the need to ensure the quality and safety of the decision in each action, usually linked to fear of making mistakes, causing harm to the patient and, consequently, likely to negatively impact students’ mental health [4–7]. Thus, it is crucial to develop, implement and evaluate strategies that enable or recreate clinical environments and clinical decision scenarios before tutored or autonomous clinical practice. These environments must recreate spaces of high realism and fidelity creating friendly learning environments and recreating emotionally safe but simultaneously challenging spaces where students can build their learning [8].

The educational strategies traditionally used in health have almost reached their highest potential, thus stimulating innovation through new andragogical strategies that support the interaction of those involved in learning in enhancing active learning and capturing the intrinsic motivation of students, directing them to the translation of knowledge, enabling more meaningful learning, and leading to greater perception of effectiveness and less likelihood of clinical error [3, 8].

In the last decades, simulation in health has emerged as a pedagogical strategy whose evidence demonstrates improved knowledge retention, instrumental, relational and communication skills, leadership and teamwork skills, and the transference of competencies [9–11].

### Clinical virtual simulation

Currently, the technological development in information and communication technologies allows to recreate patients and clinical conditions in virtual learning environments. These virtual patients are computer programs that simulate real-life clinical scenarios in which students act as health professionals, collecting the clinical history, performing the physical examination, defining the diagnosis, the intervention to be implemented, and evaluating the outcome of the clinical decision. Virtual simulation is defined as a type of simulation that places the student at the centre of decision-making, motor and/or communication skills [12].

The use of virtual patients in a virtual healthcare environment to train clinical reasoning and/or clinical decision-making skills has been defined as clinical virtual simulation (CVS) [13, 14]. Using virtual patients in education effectively improves knowledge, critical thinking, clinical reasoning, instrumental skills, self-efficacy perception, learning satisfaction, interaction and feedback, teamwork, learning experience, and realism of simulation spaces, making them emotionally safer [13, 15–24].

The increased use of clinical virtual simulation in recent years in health education was boosted during and after the COVID-19 pandemic. Despite the recognition achieved during these pandemic years, little is known about the factors that influence the acceptance by students of the use of clinical virtual simulation.

In 2016, the Nursing School of Porto began developing clinical virtual scenarios in Nursing to be integrated into the clinical virtual simulator Body Interact® (BY).

The use of clinical virtual simulation in the Nursing Degree as an andragogical strategy began in 2018–2019. Since then, studies have been conducted on usability [13, 25], knowledge retention, satisfaction and learning perception [14], the impact on learning in small groups, and the perception of curricular integration [26].

However, further investigation is needed regarding the factors that promote the adoption and use of clinical virtual simulation by students. The use of clinical virtual simulation as an integrated pedagogical strategy in a health degree implies the reorganization of the curricular plans and the introduction of andragogical strategies directed to enhancing active learning and capturing the intrinsic motivation of the student to learn [8].

### The technology acceptance model

In technology and education, the Technology Acceptance Model (TAM) is a theoretical model that predicts the acceptance of the use of technology by users [27]. This model was developed by Davis F.D. (1989) [28], to which were added the determinants of perceived utility by Davis & Venkatesh (2000) [29] and the determinants of ease of use by Venkatesh V. (2000) [30]. More recently, Venkatesh V. & Bala H. (2008) developed an integrated Technology Acceptance Model 3 (TAM3) including a structure of the individual determinants for the adoption and use of technology.

Based on the TAM3 [31], this study sought to identify the factors/determinants that predict the use of clinical virtual simulation in nursing education.

### Methodology

An observational, cross-sectional, analytical study of the use of clinical virtual simulation was carried out to answer the research question: What factors/determinants predict the acceptance and use of a clinical virtual simulator in nursing education? (Table 1).

**Table 1:** Study hypothesis

TAM Components	Hypothesis	Path
Internal constructs	H1	SU → GS
	H2	BI → SU
	H3a	PU → BI
	H3b	PEOU → PU
	H4a	PEOU → BI
External factors	H3c	SN → BI
	H3d	REL → BI
	H3e	ENJ → BI
	H3f	CSE → BI
	H3g	VOL → BI
	H4b	SN → PU
	H4c	REL → PU
	H4d	ENJ → PU
	H4e	VOL → PU
	H4f	CSE → PU
	H4g	PLAY → PU
	H5a	CANX → PEOU
	H5b	CPLAY → PEOU
	H5c	CSE → PEOU
	H5d	ENJ → PEOU
	H5e	REL → PEOU

SU–CVS use, *PU*-Perceived usefulness, *PEOU* Perceived ease of use, *CSE* Self-efficacy in the use of CVS, *PEC* Perception of external control, *CPLAY* Perceived playfulness in the use of CVS, *CANX* Anxiety with the use of CVS, *ENJ* Enjoyability associated with the use of CVS, *SN* Subjective Norm, *VOL* Voluntariness, *REL* Relevance for learning, *OUT* Evaluation result, *BI* Behaviour Intention to use, *SU*–System utilization, *GS* Global score

### Selection of participants

All students of the 2nd, 3rd, and 4th year of the Nursing Degree in the Nursing School of Porto (ESEP) (*n* = 870) were invited to participate in this study.

Considering all items related to TAM, with a count of 62 items, and applying the rule of thumb suggested by Nunnally (1978) [32] of 10 cases per variable, the recommended sample size would be 620 participants.

Following a non-probabilistic sampling methodology, a convenience sample of nursing students was selected, who voluntarily agreed to participate, and following the inclusion/exclusion criteria:

Inclusion criteria: ESEP degree students who completed attendance, with or without success, of the curricular unit Body Responses to Disease 1 (RCD 1) in the academic years 2019–2020, 2020–2021, and 2021–2022.

Exclusion criteria: ESEP degree students who have not attended the curricular unit Body Responses to Disease 1 in the academic years 2019–2020, 2020–2021, and 2021–2022, and students who obtained accreditation to the curricular unit.

In our study, the actual sample size is *n* = 619 participants. It's important to note that we do not conduct a unified analysis for all 62 items. Therefore, our sample size is similar to the calculated requirement, providing a robust foundation for the statistical analyses employed in our study.

### Ethical considerations

The ESEP’s ethics committee granted authorisation for the study 697/2022 and informed consent was obtained from all participants.

### Data collection

Data were collected through a questionnaire adapted from the Technology Acceptance Model 3 [31, 33].

The variables under study are defined in Tables 2 and 3.

**Table 2:** Determinants of perceived usefulness adapted from Venkatesh V. Bala H., 2008

Determinants	Definitions
Perceived ease of use	The degree to which a person believes that the use of CVS is effortless
Subjective norm	The degree to which a person perceives that the most important people to him/her think he/she should or should not use CVS
Image	The degree to which a person perceives that using CVS will enhance their status within their social system
Relevance for learning	The degree to which a person believes that CVS is applicable to his/her learning
Output quality	The degree to which a person believes that CVS performs its function properly
Result of learning	The extent to which a person believes the result of using CVS is tangible, observable, and communicable

**Table 3:** Determinants of perceived ease of use adapted from Venkatesh V. Bala H., 2008

Determinants	Definitions
Self-efficacy in the use of CVS	The degree to which a person believes that he/she holds the ability to perform a specific task in the CVS
Perception of external control	The degree to which a person believes that organizational and technical resources exist to support the use of CVS
Anxiety with the use of CVS	The degree of individual apprehension or fear a person faces with the possibility of using CVS
Playfulness in using CVS	The degree of cognitive spontaneity in interaction with CVS
Enjoyability associated with using CVS	The extent to which "the activity of using CVS is perceived as enjoyable, beyond the results from its use"
Objective usability	Comparison of the effort required to complete specific tasks

For the translation and validation into European Portuguese of the Technology Acceptance Model 3, authorization was granted from the authors and the following steps were carried out [33, 34]:

1. Stage I – Initial translation
  - Translation 1 from English into Portuguese performed by a professional Portuguese native translator (without knowledge of the TAM3);
  - Two translations by Portuguese native speakers, one trained in the field of Computer Sciences (without knowledge of the TAM3) (Translation 2) and one healthcare professional with experience using the TAM3 and clinical virtual simulation (Translation 3), both proficient in English.
2. Stage II—Synthesis of translations Translation 1, 2 and 3
  - Production of the Translation final version 1 (Researcher, two native Portuguese speakers, one from the health area with experience in the use of clinical virtual simulation and the TAM3 and another from the area of Computer Sciences—different from the participants in stage I);
3. Stage III—Back-translation by two English native speakers (without medical background).
4. Stage IV—Analysis by an expert group with experience in the use of the TAM3 and virtual simulation in nursing education
  - Semantic equivalence (adapted to the clinical virtual simulation);
  - Ideological equivalence;
  - Experiential equity;
  - Conceptual equivalence.
5. Stage V—Pre-test of the pre-final version with a group of 10 students who were not included in the study sample.
6. Final version—The authors waived submission of the version for evaluation and approval.

Data were collected between May and July 2022.

### Data analysis

In the initial phase, frequencies and descriptive statistics were extracted from all the collected variables. This approach allowed the analysis of the distribution of variables, evaluating the sensitivity of items (used to evaluate latent constructs) and detecting potential atypical values (outliers). Then, the Shapiro–Wilk test was used to assess the normality of the distribution of variables, and values of asymmetry and kurtosis were used to assess the degree of separation of variables from normal distribution. The main psychometric properties of the different dimensions of the TAM3 were studied. Different validity criteria (criterion and construct validity) were applied considering best practices [35] and the evaluation of their internal consistency. To evaluate the constructs internal to the TAM3 and external (individual determinants), exploratory factor analysis (EFA) was performed to define the factors associated with the constructs of the TAM3. Subsequently, confirmatory factor analysis (CFA) was employed to verify whether the items of each scale saturated the identified factors. Path analysis was used to determine the main predictors (and relevant interactions between predictors) of the intention to use clinical virtual simulation in learning, the main dependent variable to model. This analysis included other variables besides its immediate predictors, such as the perceived usefulness and perceived ease of use. Different mediation models were also developed to test whether some of the explored variables inhibited the relationship between the most relevant variables of the TAM3.

The data related to the global score variables of utilization (GS) and system utilization (SU) were extracted from the Learning Management System (LMS) of the clinical virtual simulation. The global score variable refers to the average overall evaluation obtained by the student in clinical virtual simulation utilization regarding his/her clinical decision-making skills, measured in percentage of success. The system utilization variable (SU) refers to the total number of clinical scenarios the student completed in the clinical virtual simulation.

Statistical analysis was performed using JASP, Jamovi, IBM SPSS Amos v.26, and IBM SPSS Statistics v. 26. The results are reported following the APA standards, presenting the magnitude measurements of the Cohen *d* effect (0.2 low; 0.5 medium, and 0.8 high) and considering of *P* < 0.05 as significant. In the confirmatory factor analysis, the criteria applied to evaluate the adjustment of the model were the χ2 value and its p-value, ideally nonsignificant, the CFI > 0.95, GFI > 0.90 and RMSEA > 0.03 and < 0.08 [36]. In the analysis of the convergent validity of the items internal to the TAM3, the reliability value of the construct (CR) > 0.8, the mean extracted variance (AVE) > 0.5 [36], and factor loads in inter-correlation items lower than the square root of the AVE for each construct were the adopted criteria [37].

## Results

A total of 619 Nursing students participated in this study, being 85.50% (*n* = 531) female, 35.1% (*n* = 218) 2nd-year students; 36.2% (*n* = 225) 3rd-year students; and 28.7% (*n* = 178) 4th-year students. Table 4 presents the descriptive statistics of the sample, and Table 5 the correlation matrix of age, course marks, number of completed scenarios and mean global evaluation score.

**Table 4:** Descriptive statistics of the sample

	**N**	**M**	**SD**	**Med**	**Asymmetry**	**Kurtosis**	**Min**	**Max**
Age	619	21.21	2.50	21.00	4.446	29.606	19	47
Number of completed ECTS	615	147.68	51.51	150.00	0.089	-1.241	18	240
Degree average	609	14.18	12.66	14.26	-5.116	49.549	13.2	16.6
Final mark of the course	607	13.56	14.20	14.00	0.124	-0.634	10	17

*ECTS* European Credit Transfer and Accumulation System

**Table 5:** Correlation matrix age, course marks, number of completed scenarios, and mean global evaluation score

	**Age**	**Number of completed ECTS**	**Degree average**	**Final mark of course**	**Global score of CVS use**	**Number of concluded training scenarios**
Age	–					
Number of completed ECTS	.245^**^	–				
Degree average	-0.060	0.070	–			
Final mark of course	-0.009	.093^*^	.361^**^	–		
Global score of CVS use	-.127^*^	0.049	.116^*^	.108^*^	–	
Number of concluded training scenarios	0.012	-0.029	.237^**^	.153^**^	.428^**^	–

*ECTS* European Credit Transfer and Accumulation System

^*^P < .05; ** P < .001

### Analysis of the acceptance of the use of CVS by the TAM3

Descriptive analysis of the items, followed by the exploratory factor analysis (EFA), confirmatory factor analysis (CFA), and trajectories analysis, were performed sequentially to investigate the acceptance of the use of the CVS and to identify the factors that determine the acceptance and use of the clinical virtual simulation in nursing education, as presented below.

#### Descriptive analysis of the items of the TAM3

The descriptive results of the Technology Acceptance Model 3 items, organised according to the constructs internal to the TAM and the individual determinants (constructs external to the TAM).

#### Exploratory factor analysis of the TAM

After the descriptive analysis of the data, the internal constructs associated with the Technology Acceptance Model 3 (PU-perceived usefulness; PEOU-perceived ease of use and BI-Behaviour intention to use) were analysed. Firstly, the EFA, using the Axis Factoring Main method, the Oblimin rotation method and the Kaiser Normalization criterion (KMO = 0.894, and Bartlett’s sphericity test < 0.001) were applied to analyse whether the items presented adequate factor loadings in each construct of the Technology Acceptance Model 3. The EFA allowed to identify three factors associated with the internal constructs of the Technology Acceptance Model 3 that explain 74.6% of the total variance of the data and present an adequate internal consistency (Table 6).

**Table 6:** EFA—Internal constructs of the TAM3 and Cronbach’s Alpha

	**Factor loading**	**Cronbach's Alpha**
PU1_TAM1	0.935	0.947
PU2_TAM2	0.942	
PU3_TAM3	0.924	
PU4_TAM4	0.719	
PEOU3_TAM7	0.818	0.793
PEOU4_TAM8	0.800	
PEOU1_TAM5	0.588	
PEOU2_TAM6	0.509	
BI_2_TAM41	0.992	0.955
BI_3_TAM42	0.934	
BI_1_TAM40	0.875	

*PU* Perceived usefulness, *PEOU* Perceived ease of use, *BI* behaviour Intention to use

Then, EFA was performed with the items of individual determinants of the TAM3 (e.g., CSE-Self-efficacy in the use of CVS; PEC-Perception of external control; CPLAY-Perceived playfulness in the use of CVS; CANX-Anxiety with the use of CVS; ENJ-Enjoyability associated with the use of CVS) to analyse whether the items revealed adequate factor loadings in each individual determinants of the TAM3. In the EFA, the principal axis factoring method was used with the Oblimin rotation, and the Kaiser Normalization criterion for factor extraction. To analyse the adequacy of the data to perform the EFA, the Kaiser–Meyer–Olkin test (KMO) was performed considering a value greater than 0.8 as meritorious [38] and the Bartlett’s Sphericity test *p* < 0.05 to test the relationship between the variables (KMO = 0.879; Bartlett’s sphericity test < 0.001). The EFA allowed to identify ten factors that explained 59.4% of the total variance of the data. However, items OUT_2_TAM34; CANX_1_TAM13; CPLAY_4_TAM47; PEC_4_TAM12; SN_3_TAM22; SN_4_TAM23; CPLAY_4_TAM47; PEC_4_TAM47; PEC_4_TAM12; SN_TAM23; SN_4_TAM3; SN_TA3; SN_2_TA3; SN_TAM3; SN_TAM3; SN_TAM3; SN_TAM3; SN_TAM3; SN_TAM3; SN_TAM33; SN_2_TAM3; SN_TAM3; SN_TAM3; SN_TAM3; SN_TAM_TAM3; SN_TAM3; SN_TAM33; SN_TAM3; SN_TAM3 presented factor loadings < 0.3 [36] or cross factor loadings in more than one factor, so they were excluded from the analysis. We performed a second EFA excluding the problem items described (KMO = 0.867; Bartlett’s sphericity test < 0.001). This analysis allowed to identify eight factors that explain 68.64% of the variance of the data. However, we verified that items PEC_2_TAM10, PEC_3_TAM11, and PEC_1_TAM9 had factor loadings < 0.3 or cross-factor loadings in more than one factor, so they were excluded from the analysis. Excluding these items, we performed a new EFA (KMO = 0.856; Bartlett’s Sphericity test < 0.001) that identified 7 factors, explaining 63.1% of the total variance of the data and presenting adequate internal consistency (Table 7).

**Table 7:** EFA—Constructs external to the TAM3 and Cronbach’s Alpha

		**Factor loading**	**Cronbach's Alpha**
REL	RES_3_TAM38	0.883	0.937
	RES_2_TAM37	0.881	
	REL_2_TAM31	0.691	
	REL_1_TAM30	0.674	
	REL_3_TAM32	0.637	
	OUT_1_TAM33	0.632	
	OUT_3_TAM35	0.617	
	RES_1_TAM36	0.455	
CANX	CANX_3_TAM15	0.902	0.860
	CANX_4_TAM16	0.765	
	CANX_2_TAM14	0.712	
CSE	CSE_2_TAM51	0.712	0.757
	CSE_3_TAM53	0.686	
	CSE_4_TAM55	0.643	
	CSE_1_TAM49	0.574	
VOL	VOL_2_TAM25	0.872	0.766
	VOL_3_TAM26	0.643	
	VOL_1_TAM24	0.559	
SN	SN_1_TAM20	-0.879	0.871
	SN_2_TAM21	-0.834	
CPLAY	CPLAY_1_TAM44	0.787	0.696
	CPLAY_3_TAM46	0.677	
	CPLAY_2_TAM45	0.558	
ENJ	ENJ_3_TAM19	0.908	0.929
	ENJ_1_TAM17	0.871	
	ENJ_2_TAM18	0.738	

*REL* Relevance for learning, *CANX* Anxiety with the use of CVS, *CSE* Self-efficacy in the use of CVS, *VOL* Voluntariness, *SN* Subjective Norm, *CPLAY* Perceived playfulness in the use of CVS, *ENJ* Enjoyability associated with the use of CVS

#### Confirmatory factor analysis of the TAM

**Fig. 1:**
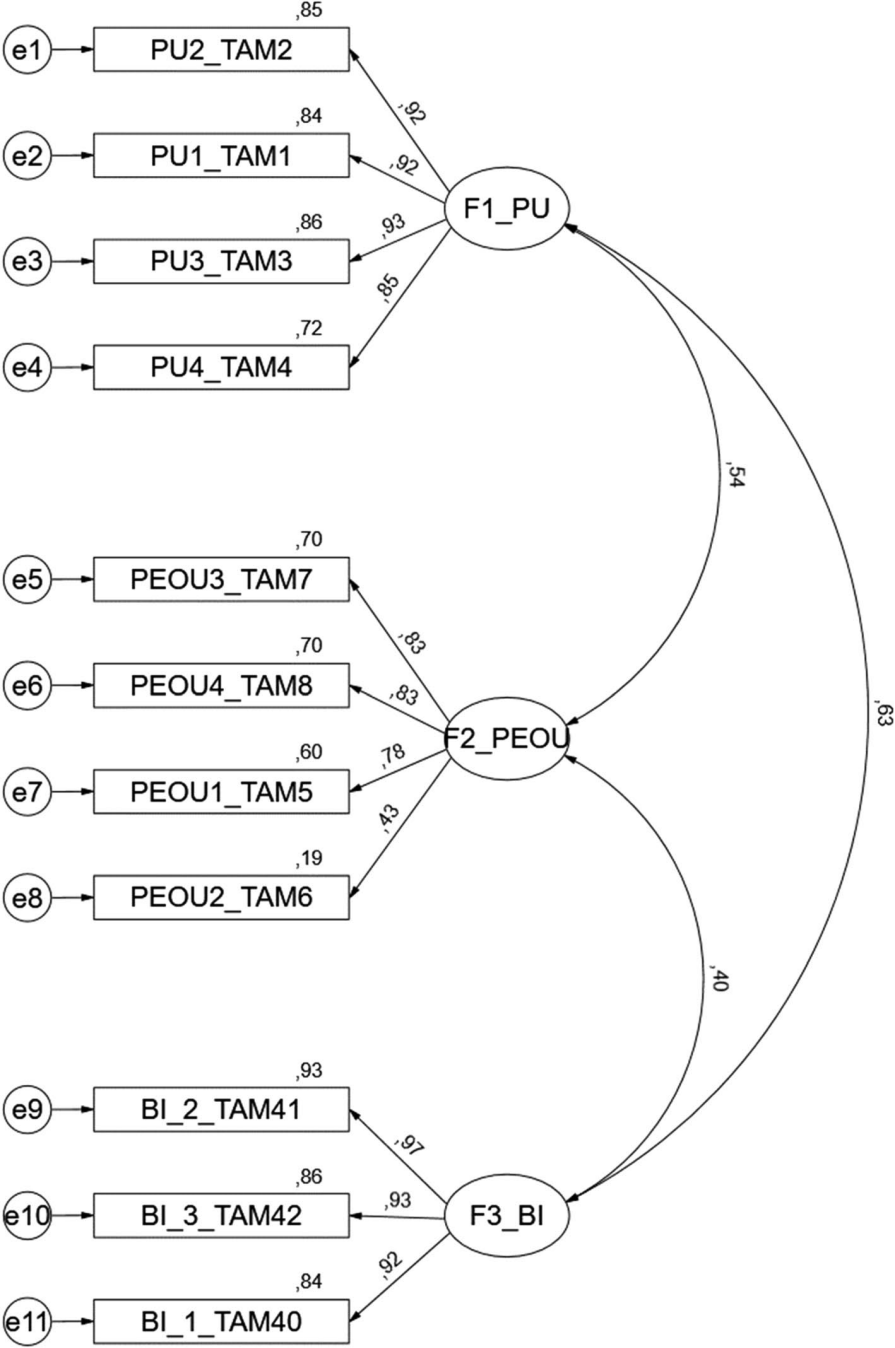
CFA model of factors internal to the TAM3. Footnote: F1-PU-Perceived usefulness; F2-PEOU-Perceived ease of use; F3-BI-Behaviour intention to use

Once the factorial structure of the Technology Acceptance Model 3 was defined for this study, a confirmatory factor analysis (CFA) was performed to validate the constructs internal to the Technology Acceptance Model 3 (Perceived usefulness, Perceived ease of use, and Behaviour intention to use), although ideally, this analysis requires a new sample. The results revealed acceptable adequacy of the model (Fig. 1) [(χ2(41) = 178, *P* < 0.001, CFI = 0.976, PCFI = 0.727, GFI = 0.948; PGFI = 0.589, RMSEA = 0.075; *p*(rmsea 0.05) < 0.001)]. Table 8 presents the results of the construct’s validity.

**Table 8:** CFA Construct validity results of internal factors to the TAM3

	**Factor loading**	**CR**	**AVE**
PU1_TAM1	0.92	0.947	0819
PU2_TAM2	0.92		
PU3_TAM3	0.93		
PU4_TAM4	0.85		
PEOU3_TAM7	0.83	0.820	0.545
PEOU4_TAM8	0.83		
PEOU1_TAM5	0.78		
PEOU2_TAM6	0.43		
BI_2_TAM41	0.97	0.956	0.878
BI_3_TAM42	0.93		
BI_1_TAM40	0.92		

*PU* Perceived utility, *PEOU* Perceived ease of use, *BI* Intention to use

**Fig. 2:**
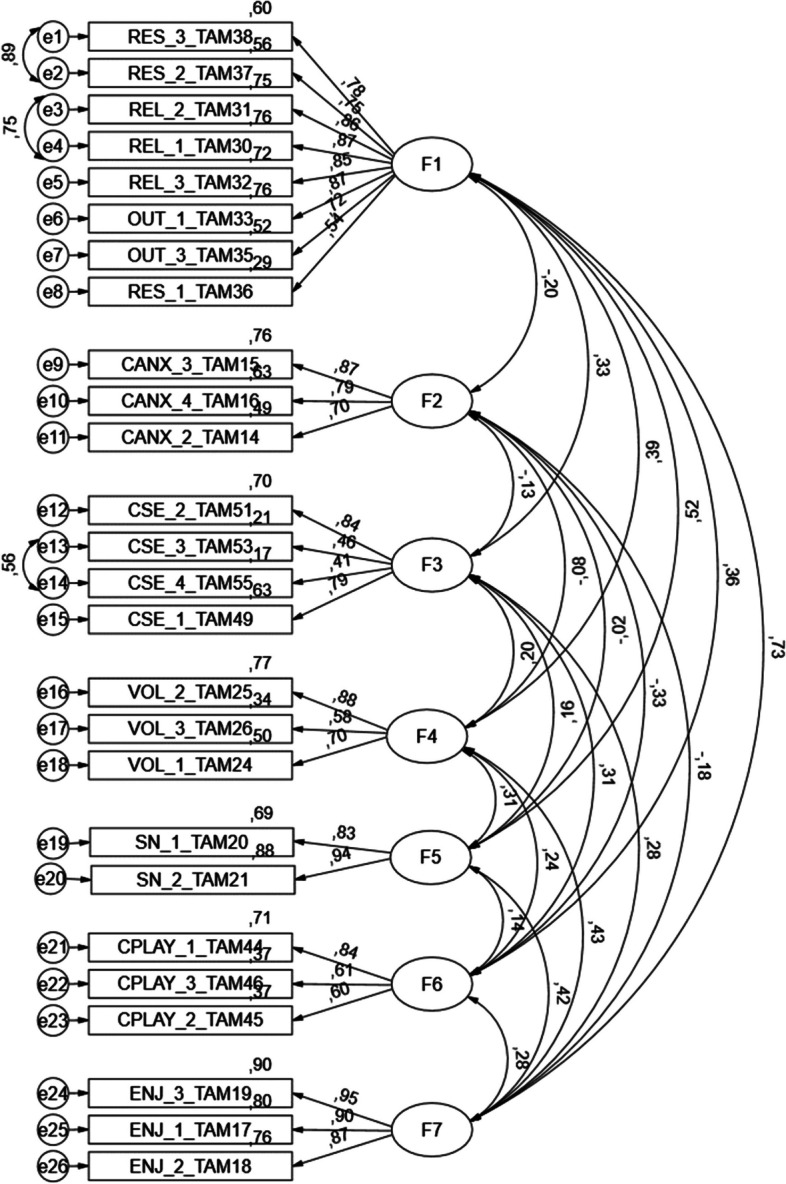
CFA model of the individual determinants of TAM 3. Footnote: F1-REL-Relevance to learning; F2—CANX—Anxiety with the use of CVS; F3—CSE—Self-efficacy in the use of CVS; F4—VOL—Voluntariness; F5—SN—Subjective Norm; F6—CPLAY—Perceived Playfulness in the use of CVS; F7—ENJ—Enjoyability associated with the use of CVS

The analysis of Table 9 shows the appropriateness of convergent and discriminant validity of the proposed model (Fig. 1). CFA was performed to validate the constructs associated with the determinants external to the Technology Acceptance Model 3 (REL-Relevance for learning; CANX-Anxiety with the use of CVS; CSE-Self-efficacy in the use of CVS; VOL-Voluntariness; PEC-Perception of external control; SN-Subjective Norm; CPLAY-Perceived playfulness in the use of CVS; ENJ-Enjoyability associated with the use of CVS) (Fig. 2). The results showed an acceptable fit to the model [(χ2(275) = 621, *P* < 0.001, CFI = 0.949, PCFI = 0.803, GFI = 0.888; PGFI = 0.695, RMSEA = 0.057; *p*(rmsea 0.05) = 0.025)]. Table 10 shows the construct validity results, with all items, except CSE_4_TAM55, presenting factorial loads greater than 0.5, revealing an acceptable convergent validity [36].

**Table 9:** Inter-construct correlation matrix of the internal items of the TAM3 diagonally, construct reliability (CR) and mean extracted variance (AVE)

		**Latent Constructs**				
Latent Constructs		**CR**	**AVE**	**1**	**2**	**3**
PU (1)		0.947	0.819	**0.905**		
PEOU (2)		0.82	0.545	0.542**	**0.738**	
BI (3)		0.956	0.878	0.632**	0,395**	**0.937**

*PU* Perceived usefulness, *PEOU* Perceived ease of use, *BI* Behaviour intention to use. On the diagonal are the inter-construct correlations and above, in bold, the square root values of the AVE

^**^P < .001

**Table 10:** CFA Construct validity results of individual determinants to the TAM3

		**Factor loading**	**CR**	**AVE**
REL	RES_3_TAM38	0.78	0.928	0.621
	RES_2_TAM37	0.75		
	REL_2_TAM31	0.86		
	REL_1_TAM30	0.87		
	REL_3_TAM32	0.85		
	OUT_1_TAM33	0.87		
	OUT_3_TAM35	0.72		
	RES_1_TAM36	0.54		
CANX	CANX_3_TAM15	0.87	0.835	0.629
	CANX_4_TAM16	0.79		
	CANX_2_TAM14	0.70		
CSE	CSE_2_TAM51	0.84	0.733	0.429
	CSE_3_TAM53	0.46		
	CSE_4_TAM55	0.41		
	CSE_1_TAM49	0.79		
VOL	VOL_2_TAM25	0.88	0.769	0.533
	VOL_3_TAM26	0.58		
	VOL_1_TAM24	0.70		
SN	SN_1_TAM20	0.83	0.879	0.784
	SN_2_TAM21	0.94		
CPLAY	CPLAY_1_TAM44	0.84	0.731	0.482
	CPLAY_3_TAM46	0.61		
	CPLAY_2_TAM45	0.60		
ENJ	ENJ_3_TAM19	0.95	0.932	0.821
	ENJ_1_TAM17	0.90		
	ENJ_2_TAM18	0.87		

*REL* Relevance for learning, *CANX* Anxiety with the use of CVS, *CSE* Self-efficacy in the use of CVS, *VOL* Voluntariness, *SN* Subjective Norm, *CPLAY* Perceived Playfulness in the use of CVS, *ENJ* Enjoyability associated with the use of CVS

The errors of the items with higher modification index values were correlated to improve the adjustment of the model. More specifically, a correlation was established between the following items: RES_3_TAM38 and RES_2_TAM37; REL_2_TAM31 and REL_1_TAM30; CSE_3_TAM53, and CSE_3_TAM55. This decision was based on the content similarity presented by the correlated items.

The convergent validity of the individual determinant items of the TAM3, a Construct Reliability (CR) revealed a value greater than 0.73, an Average Extracted Variance (AVE) greater than 0.42 [36], and factorial loadings in the inter-item correlation lower than the square root of the AVE for each construct [38], highlighted bold in Table 11 above the diagonal. The global analysis indicates the appropriateness of convergent and discriminant validity of the proposed model.

**Table 11:** Inter-construct correlation matrix, diagonal, construct reliability (CR) and mean extracted variance (AVE)

Latent Constructs	CR	AVE	1	2	3	4	5	6	7
REL (1)	0.928	0.62	**0.787**						
CANX (2)	0.835	0.629	-0.198**	**0.793**					
CSE (3)	0.733	0.429	0.328**	-0.126*	**0.655**				
VOL (4)	0.769	0.533	0.388**	-0.084	0.202*	**0.730**			
SN (5)	0.731	0.482	0.524**	-0.018	0.157**	0.305**	**0.694**		
CPLAY (6)	0.932	0.821	0.364**	-0.326**	0.305**	0.237**	0.147*	**0.906**	
ENJ (7)	0.879	0.784	0.729**	-0.18*	0.277**	0.426**	0.418**	0.277**	**0.885**

*REL* Relevance for learning, *CANX* Anxiety with the use of CVS, *CSE* Self-efficacy in the use of CVS, *VOL* Voluntariness, *SN* Subjective Norm, *CPLAY* Perceived Playfulness in the use of CVS, *ENJ* Enjoyability associated with the use of CVS. On the diagonal are the inter-construct correlations and above, in Bold, the values of the square root of the AVE

^*^P < .05; **P < .001

#### Trajectory analysis

In the proposed model, the items’ global average score of evaluation of the use of the virtual simulator (GS) and those related to the use of clinical virtual simulation (SU) were not considered (Table 12), given the high number of missing values (higher than 30%) that negatively influenced data quality and weakened the proposed model. The high number of missing values is justified by the participation in this sample of 4th-year students who attended the curricular unit Body Responses to Disease 1 in 2019–2020, an academic year in which it was not possible to extract the variables per student from the LMS.

**Table 12:** Descriptive statistics of the constructs of the TAM3

	**N**	**M**	**Med**	**Mo**	**SD**	**Asymmetry**	**Kurtosis**	**Min**	**Máx**
PU	619	5.34	5.50	6.00	1.19	-0.83	0.62	1.00	7.00
PEOU	619	4.74	4.75	4.75	1.07	-0.41	0.18	1.00	7.00
BI	619	5.21	5.67	7.00	1.63	-0.83	-0.05	1.00	7.00
REL	619	5.07	5.25	6.00	1.18	-0.69	0.35	1.00	7.00
CANX	619	1.50	1.00	1.00	1.07	2.72	7.80	1.00	7.00
CSE	606	6.72	6.67	5.00	1.61	-0.08	-0.27	1.00	10.00
VOL	619	4.77	5.00	7.00	1.61	-0.48	-0.50	1.00	7.00
SN	617	4.14	4.00	4.00	1.61	-0.29	-0.31	1.00	7.00
CPLAY	616	5.59	5.67	7.00	1.14	-0.73	0.27	1.00	7.00
ENJ	619	5.54	6.00	7.00	1.33	-0.99	0.76	1.00	7.00
GS	383	82.90	84.50	84.10^a^	8.43	-1.12	1.26	52.00	100.00
SU	386	31.85	31.50	43.00	16.84	0.71	1.14	1.00	102.00
Footnote: ^a^ There are several modes. The lowest value is presented									

*REL* Relevance for learning, *CANX* Anxiety with the use of CVS, *CSE* Self-efficacy in the use of CVS, *VOL* Voluntariness, *SN* Subjective Norm, *CPLAY* Perceived Playfulness in the use of CVS, *ENJ* Enjoyability associated with the use of CVS, *GS* Global score, *SU* CVS use

**Fig. 3:**
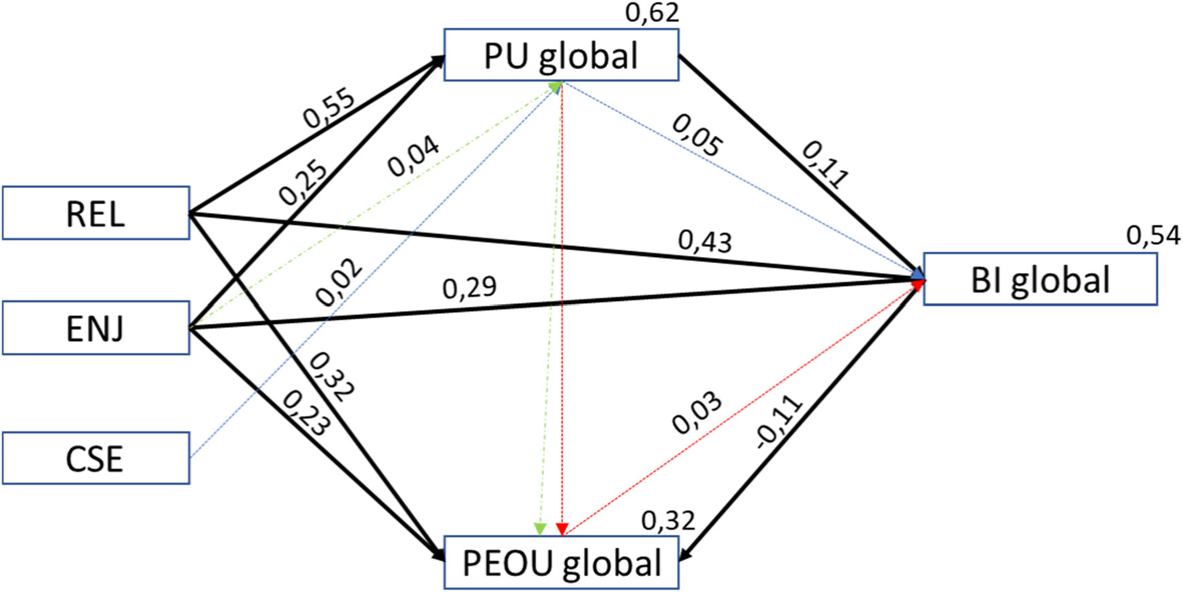
Model proposed for the acceptance of the technology for CVS (TAM3CVS_MP). Legend: REL-Relevance for learning; CSE-Self-efficacy in the use of CVS; ENJ-Enjoyability associated with the use of CVS; Black lines in Bold—direct effects; Dashed lines (blue/green and red)—indirect effects

Trajectory analysis was used to analyse the adequacy of the TAM3 adapted to assess the acceptance of the use of clinical virtual simulation. Table 13 summarises the tested hypotheses. Figure 3 represents the standardised constructs coefficients of the model and coefficients of determination (*R* ^2^) associated with the modelling of the dependent variables. The model presents values of [(χ^2^(5) = 7.22, *p* = 0.205, CFI = 0.999, PCFI = 0.111, GFI = 0.998; PGFI = 0.091, RMSEA = 0.027; *p*(rmsea 0.05) = 0.789)]. In Fig. 3, the proposed model explains a high variance of perceived usefulness (*R* ^2^ = 62%), intention to use the CVS (*R* ^2^ = 54%), and a lower variance of ease of use (*R* ^2^ = 32%).

**Table 13:** Summary of hypotheses, *β* values and results

TAM Components	Hypothesis	Path	*β*-value	*p*-value	Result
Internal constructs	H1	SU → GS			*Not tested*
	H2	BI → SU			*Not tested*
	H3a	PU → BI	0.11	0.012	Supported
	H3b	PEOU → PU	0.06	0.45	Not supported
	H4a	PEOU → BI	-0.11	0.02	Supported
External factors	H3c	SN → BI	0.55	0.096	Not supported
	H3d	REL → BI	0.43	***	Supported
	H3e	ENJ → BI	0.29	***	Supported
	H3f	CSE → BI	-0,02	0.445	Not supported
	H3g	VOL → BI	0.04	0.196	Not supported
	H4b	SN → PU	0.02	0.548	Not supported
	H4c	REL → PU	0.55	***	Supported
	H4d	ENJ → PU	0.25	***	Supported
	H4e	VOL → PU	-0.01	0.71	Not supported
	H4f	CSE → PU	0.01	0.659	Not supported
	H4g	PLAY → PU	0.02	0.982	Not supported
	H5a	CANX → PEOU	-0.05	0.49	Not supported
	H5b	CPLAY → PEOU	0.02	0.517	Not supported
	H5c	CSE → PEOU	0.1	0.008	Supported
	H5d	ENJ → PEOU	0.23	***	Supported
	H5e	REL → PEOU	0.32	***	Supported

*PU* Perceived usefulness, *PEOU* Perceived ease of use, *BI* Behaviour intention to use, *REL* Relevance for learning, *CANX* Anxiety with the use of CVS, *CSE* Self-efficacy in the use of CVS, *VOL* Voluntariness, *PEC* Perception of external control, *SN* Subjective Norm, *CPLAY* Perceived Playfulness in the use of CVS, *ENJ* Enjoyability associated with the use of CVS

^***^P < .001

Table 13 shows that the defined hypotheses related to the internal constructs and the individual determinants of the TAM3 regarding the acceptance of the clinical virtual simulation, the perceived usefulness (PU), the perceived ease of use (PEOU) and the behaviour intention to use (BI)], are influenced by Relevance for learning (REL) [REL → PU (*β* = 0.55; *P* < 0.001), REL → PEOU (*β* = 0.32; *P* < 0.001), REL → BI (*β* = 0.43; *P* < 0.001)], and Enjoyability (ENJ) [ENJ → PU (*β* = 0.25; *P* < 0.001), ENJ → PEOU (*β* = 0.23; *P* < 0.001), ENJ → BI (*β* = 0.29; *P* < 0.001)]. The remaining hypotheses were not supported in this study.

Table 14 shows the standardised indirect effects of the Model proposed for the acceptance of the technology for CVS (TAM3CVS_MP).

**Table 14:** Standardised indirect effects

	**VOL**	**CPLAY**	**CANX**	**CSE**	**ENJ**	**REL**	**SN**	**PEOU**	**PU**
PEOU									
PU		0.37	0.1	0.02*	0.044*	0.07			
BI	0.529	0.637	0.073	0.05*	0.795	0.281	0.561		0.026*

*PU* Perceived usefulness, *PEOU* Perceived ease of use, *BI* Behaviour intention to use, *REL* Relevance for learning, *CANX* Anxiety with the use of CVS, *CSE* Self-efficacy in the use of CVS, *VOL* Voluntariness, *PEC* Perception of external control, *SN* Subjective Norm, *CPLAY* Perceived playfulness in the use of CVS, *ENJ* Enjoyability associated with the use of CVS

^*^P < .05

## Discussion

The participants showed mean values of perceived usefulness (M = 5.34; SD = 1.19), ease of use (M = 4.74; SD = 1.07), and behaviour intention to use the clinical virtual simulation (M = 5.21; SD = 1.18), indicating the acceptance of the use of clinical virtual simulation in nursing education.

This study validated the use of the Technology Acceptance Model 3 adapted to clinical virtual simulation and tested the related hypotheses, showing that the model explains 62% of perceived usefulness, 32% of ease of use, and 54% of behaviour intention to use the clinical virtual simulation by nursing students. The adequacy of the model was tested by analysing the direct effects of the relationships between the internal constructs (PU → BI, *β* = 0.11, *P* = 0.012; PEOU → BI, *β* = -0.11, *P* = 0.002) and direct relations between some of the constructs internal to the TAM and the external determinants, Relevance for learning and Enjoyability. Also, the adequacy of the proposed model was determined by analysing the indirect effects of self-efficacy in the use of clinical virtual simulation (CES) on BI (*P* = 0.05) through PU (*P* = 0.02) and the indirect effect on Enjoyability (ENJ) on PU through PEOU (*P* = 0.044) and the indirect effect of PU on BI through PEOU (*P* = 0.026).

In sum, regarding the technology acceptance model for clinical virtual simulation, the internal constructs that predicted the intention to use were perceived usefulness and perceived ease of use. However, perceived ease of use emerges as new its inverse relationship with the behaviour intention to use. This fact finds no parallel in the evidence [33, 39–43]. This data points out that the responses expressed by the students are not related to the ease of use inherent to technology but rather to the cognitive performance necessary for the resolution of clinical scenarios and the training of clinical decision-making skills. Thus, the greater the perception of ease of use in a perspective of greater competence in the clinical decision-making process, the lower the intention to use the clinical virtual simulation. These data also reveals the need for clinical scenarios to present an increasing level of complexity according to the development of clinical decision-making skills.

In the proposed model, Relevance for learning (REL) and Enjoyability (ENJ) were the external constructs that best predicted perceived usefulness, ease of use, and behaviour intention to use clinical virtual simulation. This is in line with some of the findings of a meta-analysis by Rosli M.S et al. (2022) [43], the study of the adequacy of the Technology Acceptance Model 3 to virtual reality by Jiang, M et al. [39], and the study on the acceptance of computer games as an educational strategy, where Relevance for learning was also identified as one of the best predictors of perceived usefulness and/or ease of use by Lemay, D. J et al. [41].

The decision on the behaviour intention to use the clinical virtual simulation should consider three indirect effects identified:

- The self-efficacy in the use of the clinical virtual simulation (CVS) indirectly predicts the behaviour intention to use the CVS through the moderation of perceived usefulness. This emphasises the need to optimise the support to students with less perceived self-efficacy in the use of CVS;
- Enjoyability predicts ease of use of CVS through moderation of perceived usefulness. This fact points to the perception of enjoyability having a positive influence on the perceived usefulness, which positively influences the ease of use of the CVS. Also, this demonstrates that the increased Enjoyability associated with use can help overcome some of the complexity perceived by the student in the use of CVS;
- The perceived usefulness predicts the behaviour intention to use CVS through moderation of perceived ease of use. These data point out that a greater utility perceived by the student in the use of CVS helps overcome some of the complexity perceived in the use of CVS in clinical reasoning training.

The analysis of the descriptive data associated with each construct internal to the TAM3 and the individual determinants showed average scores ranging between 4.14–5.59, except for Anxiety related to the use of the CVS, with an average score of 1.5, indicating its low perception by the students. The average self-efficacy score of the use of CVS (M = 6.72) is explained by the fact that it is evaluated on a 10-point Guttman scale. The lowest average score observed is for the subjective norm, indicating that it is not the influence of other people, particularly teachers, that determined the use of CVS by students. Regarding the voluntary use of CVS, data should be interpreted with caution since items TAM25 (M = 4.71) and TAM26 (M = 4.34) revealed a low perception of obligation perceived by the student to use the CVS, while item TAM24 (M = 5.24) showed a higher value associated to the voluntary use of the CVS. Regarding the ease of use, item TAM6 (M = 4.06) revealed that the use of CVS did not require much effort, as opposed to item TAM7 (M = 5.10), expressing the ease of use.

These are innovative results as they highlight the positive influence that the relevance attributed to learning and development of clinical reasoning and clinical decision-making skills (Learning Relevance) have on the perception of ease of use, perceived usefulness in the use of CVS, and the effective use of CVS. These results are associated with the relevance attributed by the student who views the use of CVS as linked to the triggering of emotions such as enjoyability, a fact previously determined as a variable to adjust the use of technology by Venkatesh V. & Bala H. (2008) [31] but still little explored by Kim, S et al. [44]. This study shows that the use of CVS, ease of use, and usefulness are influenced by the positive representation that the student has of the contribution to their training as a future nurse. Also, these study results demonstrate that the use of CVS creates a playful context that helps students learn actively in a friendly environment, bringing aspects of gamification that help them set goals, get scores, and compare results between students [44, 45] while simultaneously anticipating clinical challenges. Using game elements added to the CVS contributes to developing intrinsic motivation [46] and satisfaction with the learning process [8].

The use of CVS promotes students’ active learning and the capture of their intrinsic motivation through facilitated access to pedagogical resources according to the pacing and learning preferences of the students. This learning environment promotes autonomy, the development of effectiveness and belonging to a learning community, an environment in which the teacher is a facilitator of the learning process, and the student learns while having fun [46–48]. It can be argued that the motivation under analysis can be extrinsic [46] because it uses a perception of locus of internal causality associated with an integrated regulation process by anticipating the results that students may achieve, a fact represented by the construct Relevance for learning (REL). However, the construct Enjoyability clarifies the existence of intrinsic motivation based on interest and student satisfaction with the learning process using the CVS [46]. Thus, this study showed that a personal determinant of the student (REL) and an adjustment determinant (ENJ) are central to the use of CVS in nursing education.

Currently, the acceptance and adoption of CVS in Nursing education, in this context, goes beyond external variables to the student that may determine the adoption of CVS, for example, the influence of teachers and significant people or individual determinants related only to characteristics of CVS, for example, the effectiveness in the use of CVS, anxiety associated with the use of CVS, and playfulness. This study revealed that the current characteristics of pre-graduation students, who are digital natives [49], lead to features related to the use of technology that may be overcome by the nature of the learning outcomes anticipated by students.

In sum, this study produced interesting outcomes for nursing education in this context, affirming that the use of CVS in learning is directly determined by students’ perceived relevance and enjoyability. This positively influences the usefulness and perceived ease of use and consequently the behaviour intention to use the CVS. Perceived Self-efficacy indirectly predicted the behaviour intention to use CVS through moderation by perceived usefulness.

The results of this study require careful interpretation because they only represent a single context of nursing degree education and were implemented in one of the curriculum units of the syllabus. Notwithstanding, this study presents data that can support educators in the health field in making decisions or developing new studies, overcoming some of the limitations of this study.

### Study limitations

The main limitations of this study were the use of the same sample of students to perform the EFA and CFA.

Another identified limitation was not having included the construct of the attitude referred to in other studies. This option was based on the expectation of having data related to the behaviour from the evaluation and use of the CVS. Also, the lack of data for the entire sample regarding the Global Scores of use and the number of completed scenarios per student conditioned the potential of the presented model. Thus, using this model in samples with these available data is recommended. This limitation stems from the fact that CVS is still little used as an andragogical strategy in health education.

## Conclusion

This study provided noteworthy contributions to propose a technology acceptance model for clinical virtual simulation (TAM3CVS_MP), identifying the factors determining the acceptance and use of clinical virtual simulation by nursing students.

The results showed the potential of clinical virtual simulation as a pedagogical strategy to capture students' intrinsic motivation to develop active and optimised learning while potentiating their skills.

Integrating clinical virtual simulation as an andragogical strategy in nursing education curriculums needs to rely on higher education institutions and teachers, investment in training, technology and time for reflection, discussion, and analysis. However, this study provides information that can support the decision and shape the implementation strategy.

Furthermore, this study evidences the importance of teachers’ and institutional decision-makers’ attention to students' perception of relevance for learning, enjoyability, and perceived self-efficacy associated with the use of clinical virtual simulation during conceptualisation, design, and implementation processes.

### Supplementary Information

**Additional file 1.** Descriptive analysis of the TAM item.

## Data Availability

The data that support the findings of this study are available from the corresponding author but restrictions apply to the availability of these data, which were used under license from the Nursing School of Porto (Portugal) for the current study, and so are not publicly available. Data are, however, available from the authors upon reasonable request and with permission from Nursing School of Porto (Portugal).

## Abbreviations

AVE: Average extracted variance
BI: Behaviour intention to use
CANX: Anxiety with the use of CVS
CFA: Confirmatory factor analysis
CPLAY: Playfulness in the use of CVS
CR: Construct reliability
CSE: Self-efficacy in the use of CVS
CVS: Clinical virtual simulation
ECTS: European credit transfer and accumulation system
EFA: Exploratory factor analysis
ENJ: Enjoyability associated with the use of CVS
ESEP: Nursing School of Porto
GS: Global score
HEIs: Higher education institutions
IMG: Image
KMO: Maiser-Meyer-Olkin test
LMS: Learning management system
OU: Objective usability
OUT: Evaluation result
PEC: Perception of external control
PEOU: Perceived ease of use
PU: Perceived usefulness;
RCD 1: Body responses to disease 1 course
REL: Relevance for learning
RES: Learning relevance
SN: Subjective norm
SU: CVS use
TAM: Technology acceptance model
VOL: Voluntariness
