# Supplementary material for: Clinical virtual simulation: predictors of user acceptance in nursing education
Source: BMC Med Educ. 2024 Mar 16;24:299. doi: 10.1186/s12909-024-05154-2 (PMC10943828; doi:10.1186/s12909-024-05154-2)
Supplement: Supplementary file 1 — Additional file 1. Descriptive analysis of the TAM item. [file 12909_2024_5154_MOESM1_ESM.docx]

Appendix 1- Descriptive analysis of the TAM items
